# Different Effects of Vitamin C-Based Supplements on the Advance of Linseed Oil Component Oxidation and Lipolysis during In Vitro Gastrointestinal Digestion

**DOI:** 10.3390/foods11010058

**Published:** 2021-12-27

**Authors:** Bárbara Nieva-Echevarría, Encarnación Goicoechea, Patricia Sopelana, María D. Guillén

**Affiliations:** Food Technology, Lascaray Research Center, Faculty of Pharmacy, University of the Basque Country (UPV/EHU), 01006 Vitoria-Gasteiz, Spain; barbara.nieva@ehu.eus (B.N.-E.); encarnacion.goicoechea@ehu.eus (E.G.); patricia.sopelana@ehu.eus (P.S.)

**Keywords:** vitamin C supplements, ascorbic acid, ascorbyl palmitate, in vitro gastrointestinal digestion, flaxseed oil, lipid oxidation, lipolysis, oxygenated alpha,beta-unsaturated aldehydes, ^1^H NMR, SPME-GC/MS

## Abstract

Although widely consumed, dietary supplements based on Vitamin C contain high doses of this compound, whose impact on lipid oxidation during digestion needs to be addressed. Therefore, the effect of seven commercial supplements and of pure l-ascorbic acid and ascorbyl palmitate on linseed oil during in vitro gastrointestinal digestion was tackled. The advance of lipid oxidation was studied through the generation of oxidation compounds, the degradation of polyunsaturated fatty acyl chains and of gamma-tocopherol, by employing Proton Nuclear Magnetic Resonance. Supplements containing exclusively l-ascorbic acid enhanced the advance of linseed oil oxidation during digestion. This was evidenced by increased formation of linolenic-derived conjugated hydroxy-dienes and alkanals and by the generation of conjugated keto-dienes and reactive alpha,beta-unsaturated aldehydes, such as 4,5-epoxy-2-alkenals; moreover, gamma-tocopherol was completely degraded. Conversely, supplements composed of mixtures of ascorbic acid/salt with citric acid and carotenes, and of ascorbyl palmitate, protected linseed oil against oxidation and reduced gamma-tocopherol degradation. The study through Solid Phase Microextraction-Gas Chromatography/Mass Spectrometry of the volatile compounds of the digests corroborated these findings. Furthermore, a decreased lipid bioaccessibility was noticed in the presence of the highest dose of l-ascorbic acid. Both the chemical form of Vitamin C and the presence of other ingredients in dietary supplements have shown to be of great relevance regarding oxidation and hydrolysis reactions occurring during lipid digestion.

## 1. Introduction

The occurrence of lipid oxidation during gastrointestinal digestion has been evidenced in recent decades [1,2]. In this context, several works have been devoted to shedding light on the extent of this reaction during digestion and the factors affecting it, as well as on the potentially relevant role of dietary antioxidants in minimizing the negative impact of the occurrence of oxidation reactions in the gastrointestinal tract [3,4,5]. Indeed, the oxidation of food lipids under digestive conditions would lead not only to a loss of their nutritional value but also to the generation of derived products of very different nature, which could exert negative effects in the gastrointestinal tract itself and/or be absorbed through the intestinal cells and further promote diseases [6,7,8]. Thus, the generation under in vitro, ex vivo and in vivo gastrointestinal conditions of cytotoxic and genotoxic oxygenated alpha,beta-unsaturated aldehydes, such as 4-hydroxy-2-nonenal or 4,5-epoxy-2-heptenal, from polyunsaturated lipids has been reported [5,9,10,11,12,13,14]. Concomitantly, under such oxidative conditions, other minor food components of nutritional interest would also be oxidatively degraded before absorption, as reported for carotenes, catechins and tocopherols [9,15,16,17,18].

Furthermore, in the last decade, there has been a clear trend among consumers to increase the intake of antioxidant compounds, coming either from food or through the consumption of dietary supplements. Among the molecules generally considered as powerful antioxidants, there is Vitamin C or l-ascorbic acid. This compound, essential for humans, is mainly provided through the consumption of fruits and vegetables and has been reported to protect from cardiovascular disease, stroke, cancer and neurodegenerative diseases [19,20]. Moreover, oral supplements of ascorbic acid or its derivatives (ascorbyl palmitate or stearate) are being commonly consumed worldwide in the belief that increased amount of Vitamin C will provide increased resistance to oxidative stress and thus to certain diseases [20]. However, there is still a debate regarding the health benefits of Vitamin C, especially in relation to supplementation, as it is known that it may show toxic pro-oxidant activity under certain conditions [19,20,21,22,23,24]. It must be noted that Vitamin C supplementation presents at least two large differences in comparison with fruit and vegetable consumption: (i) the amount of this compound provided by the supplement is much higher than that naturally found in these foods; and (ii) the lack of other compounds with potential antioxidant activity present in fruits and vegetables together with ascorbic acid, dismissing the potential influence of an “antioxidant network” on the health benefits attributed to Vitamin C.

Concerning in vitro digestion studies performed to date, a very small number of them have investigated the effect of Vitamin C, as either ascorbic acid or ascorbyl palmitate, on the advance of lipid oxidation under simulated digestion conditions. Moreover, it must be pointed out that the concentrations studied were similar to those that could be naturally ingested during a meal, which are much lower than those reached after Vitamin C supplement intake. Even so, the reported results are inconclusive. Thus, early studies regarding the effect of l-ascorbic acid on in vitro digestion of turkey meat with human gastric fluids evidenced a lower formation of hydroperoxides when present at 0.25 mM [15]. More recently, Rysman et al. [25] detected significantly lower concentration of Thiobarbituric Acid Reactive Substances (TBARS) in the in vitro gastrointestinal digests of sausages containing 0.05% of sodium ascorbate, compared with the control. In line with these results, during in vitro gastrointestinal digestion of low-fat beef meat a significant inhibition of the generation of malondialdehyde, hexanal and 4-hydroxy-2-nonenal was reported in the presence of l-ascorbic acid at concentrations ranging from 0.7 to 3.0 mM, approximately [11]. However, in this latter study, the opposite effect was observed in the case of high-fat beef meat in vitro digestion. Likewise, Larsson et al. [12] evidenced that the formation of malondialdehyde and 4-hydroxy-2-hexenal increased in the presence of ascorbic acid at 0.085 mM during cod liver oil dynamic in vitro digestion. In contradiction with the above-mentioned studies, Tarvainen et al. [26] did not observe significant differences in the amount of oxidized compounds after in vitro gastrointestinal digestion of fresh rapeseed oil in the absence or presence of l-ascorbic acid at ≈0.5 and 4.8 mM, as well as in the presence of ascorbyl palmitate at ≈0.2 and 2.1 mM.

Vitamin C doses provided through supplements (e.g., 400–2500 mg/dose) significantly exceed the Population Reference Intake of Vitamin C recommended by the European Food Safety Authority, which is 95–110 mg/day for adults [27]. As this vitamin is hydrosoluble, it is claimed that if ingested in certain excess, it will be excreted from the body via urine without major health consequences. Despite some of the aforementioned studies [11,12] where the generation of toxic lipid oxidation products under digestion conditions has been shown to be enhanced by low doses of ascorbic acid, there is a lack of studies dealing with the effect of high doses of Vitamin C on the oxidation reactions occurring in the gastrointestinal tract. Therefore, it is considered of great interest to investigate what could happen when small amounts of food lipids are ingested together with Vitamin C supplements. For this purpose, linseed oil, a highly polyunsaturated edible oil prone to oxidation, will be submitted to in vitro gastrointestinal digestion in the presence of seven commercial oral supplements of varied complexity, containing different chemical forms of Vitamin C (acid, salt or its lipophilic derivative ascorbyl palmitate) and different doses (400–2500 mg/dose), as well as in the presence of pure l-ascorbic acid and ascorbyl palmitate. Proton Nuclear Magnetic Resonance (^1^H NMR) and Solid Phase Microextraction-Gas Chromatography/Mass Spectrometry (SPME-GC/MS) will be used to study simultaneously a broad range of linseed oil oxidation products potentially formed during in vitro gastrointestinal digestion in the presence of supplements and pure l-ascorbic acid and ascorbyl palmitate. Furthermore, ^1^H NMR will also be employed to analyze the potential changes occurring during digestion in the concentration of gamma-tocopherol, a minor component of linseed oil considered as potential antioxidant, which could be degraded if involved in oxidation reactions. Finally, differences, if any, in the degradation of polyunsaturated fatty acyl chains and in the lipolysis level reached among the digests will also be investigated through ^1^H NMR where possible.

## 2. Materials and Methods

### 2.1. Dietary Supplements Based on Vitamin C, Edible Oil and Pure Compounds Employed

Seven oral dietary supplements based on Vitamin C (VC1–7) of different commercial brands and showing different formulation and delivery form were acquired in local supermarkets. They were selected in order to cover a broad range of commercially available food supplements of this kind of vitamin. Their composition according to the label is shown in Table 1.

VC1 contains exclusively l-ascorbic acid powder, its recommended dose being 2500 mg/day. VC2, VC3 and VC4 are capsules containing 1500, 1000 and 500 mg of l-ascorbic acid, respectively, and also additional components, such as certain bulking, anti-caking and coating agents. VC5 consists of effervescent tablets containing 1000 mg of l-ascorbic acid and other ingredients, such as citric acid, carotenes and sweeteners, among others. VC6 are drinkable vials containing 1000 mg of liposomal encapsulated sodium ascorbate (salt), in addition to other ingredients, such as orange juice concentrate, citric acid, emulsifiers for the formation of liposomes, gellan gum, sweeteners or beta-carotene. VC7 contains 400 mg of ascorbyl palmitate together with bulking agents in a cellulose capsule.

The edible oil employed for in vitro digestion experiments was a commercial virgin linseed oil (L). This oil was selected because it is especially prone to oxidation due to its high content of polyunsaturated linolenic (C18:3n-3) acyl groups.

Bearing in mind the potential influence of other components in the dietary supplements (included as ingredients or coming from capsules’ composition; see Table 1), for comparative purposes, pure l-ascorbic acid (CAS 50-81-7, purity: 99%, Merck KGaA, Darmstadt, Germany) and ascorbyl palmitate (CAS 137-66-6, purity: ≥98%, CarboSynth Ltd., Berkshire, UK) were also purchased and used for in vitro digestion experiments. In the case of ascorbyl palmitate, the amount of 400 mg was selected (AP) because it was the amount present in VC7 supplement, according to the manufacturer. Taking this as a reference, the following quantities of l-ascorbic acid were added to linseed oil samples: 170 mg (Asc1) (equimolar amount of 400 mg of ascorbyl palmitate) and 400 mg (Asc2).

### 2.2. In Vitro Gastrointestinal Digestion

The in vitro digestion model employed is a semi-static procedure that mimics digestive processes occurring in the mouth, stomach and duodenum by sequentially adding digestive juices (saliva, gastric juice, duodenal juice and bile juice) to the sample, while incubated at 37 ± 2 °C and rotated head-over-heels throughout the duration of the experiment. The transit times employed for oral, gastric and intestinal steps were 5 min, 2 h and 4 h, respectively. This in vitro procedure was initially developed by Versantvoort et al. [28] and slightly modified later to reach a higher degree of lipolysis, similar to that occurring in vivo [29]. In the Supplementary Materials Section, a detailed description of the digestion procedure can be found (Section S1), as well as of the composition of the simulated digestive juices (see Table S1).

Aliquots of linseed oil (0.5 g) were submitted in triplicate to in vitro gastrointestinal digestion in the absence and in the presence of each one of the aforementioned dietary supplements (VC1–7) and of pure compounds AP, Asc1–2. The in vitro digests obtained from linseed oil in the absence of supplements or pure compounds were named DL. Those obtained from co-digestion of linseed oil with supplements were named DLVC1–7 and with pure compounds, DLAP, DLAsc1 and DLAsc2. Taking into account the total volume of digestive juices employed during the gastric and intestinal steps (18 and 38 mL, respectively—see Section S1 of the Supplementary Materials) and the supplement dosage (see Table 1), the concentrations of Vitamin C during the gastric and intestinal steps were estimated to be 54 and 25 mM in the case of DLAsc1, DLAP and DLVC7; 126 and 60 mM for DLAsc2; 158 and 75 mM for DLVC4; 203 and 118 mM for DLVC6; 315 and 149 mM for DLVC3 and DLVC5; 473 and 224 mM for DLVC2; and 789 and 374 mM for DLVC1.

### 2.3. Lipid Extraction for Further Analysis through ^1^H NMR

Lipids of the in vitro linseed oil digests were extracted following the same methodology as in previous studies [29,30]. A liquid–liquid extraction of 38 mL of digest was performed, involving three extraction stages, each with 20 mL of dichloromethane (CH_2_Cl_2_, HPLC grade, Merck KGaA, Darmstadt, Germany). Afterward, to ensure a complete protonation of fatty acids and/or the dissociation of the potential salts formed, the remaining water phase was acidified to pH ≈ 2 with HCl (37%, Merck KGaA), and a second extraction was carried out (3 × 20 mL). All CH_2_Cl_2_ extracts of each sample were mixed, and the solvent was eliminated by means of a rotary evaporator under reduced pressure at room temperature and protected from light exposure. The concentrated lipid residue was stored at −80 °C until its analysis through ^1^H NMR.

### 2.4. Study through ^1^H NMR of the Starting Oil and of the Lipid Extracts of the Digests

#### 2.4.1. Preparation of the Lipid Samples and Operating Conditions for Spectra Acquisition

The ^1^H NMR spectra of linseed oil (L) and of the lipid extracts of several digests (DL, DLVC1–7, DLAsc1–2, DLAP) were acquired in duplicate using a Bruker Avance 400 spectrometer operating at 400 MHz. Since in vitro digestion experiments were carried out in triplicate, a total *n* of 6 was obtained for each sample. As for sample preparation, 200 μL of oil or of the digested lipid extract was mixed in a 5 mm diameter ^1^H NMR tube with 400 μL deuterated chloroform (CDCl_3_), which contained 0.2% of non-deuterated chloroform and a small proportion of tetramethylsilane (Euroisotop, Paris, France). For each tube, two experiments were carried out: a standard single-pulse ^1^H NMR experiment and a NOESYGPPS experiment. This latter consists of a one-dimensional ^1^H NMR pulse sequence with selective suppression of the strong proton signals of linseed oil acyl groups A, B, C, E, F, G, H and T (see Table S2 in Supplementary Materials Section); this increases the sensitivity regarding the spectral region, ranging from 5.8 to 9.8 ppm, and thus enables the detection and quantification of certain minor compounds that are not present in sufficient concentrations to be detected by standard pulse experiment [31]. The acquisition parameters of both experiments were the same as those used in previous studies [31,32]. The relaxation delay and acquisition time allow a complete relaxation of the protons, the signal areas thus being proportional to the number of protons that generate them, making it possible to use them for quantitative purposes.

**Figure 1:**
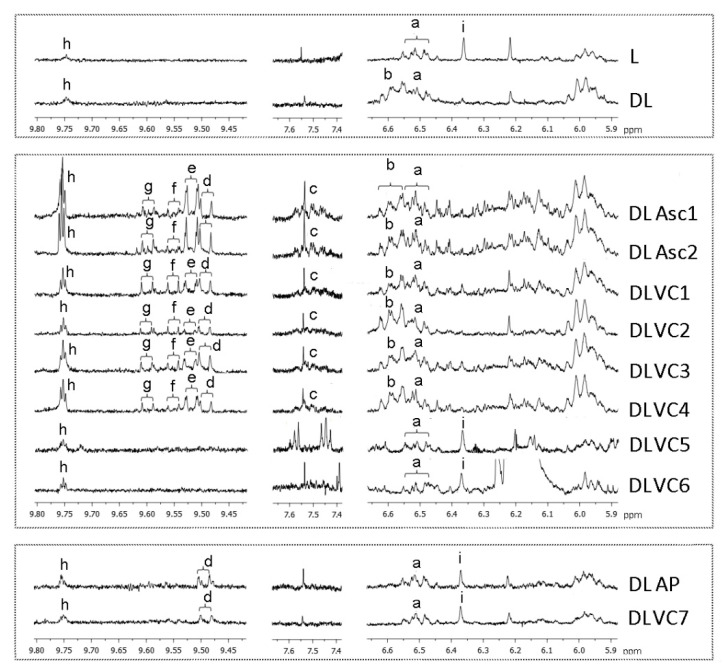
Enlargement of certain regions of the multisuppressed ^1^H NMR spectrum of linseed oil (L) and of the corresponding lipid extracts obtained after in vitro digestion in the absence (DL) and in the presence of supplements and pure l-ascorbic acid and ascorbyl palmitate (DLVC1–7, DLAsc1–2, DLAP). The signal letters **a** to **h** correspond with those given in Table 2.

The ^1^H NMR spectra shown in Figure 1 were plotted at a fixed value of absolute intensity to be valid for comparative purposes and processed using MNova program (Mestrelab Research, Santiago de Compostela, Spain). Assignment of the chemical shifts and multiplicities of the ^1^H NMR signals in deuterated chloroform (CDCl_3_) are provided in Table 2.

#### 2.4.2. Quantitative Determinations from ^1^H NMR Spectral Data

Several quantitative determinations were performed by using spectral data obtained from both the recorded standard and multisuppressed ^1^H NMR spectra because, as previously commented, the area of each ^1^H NMR spectral signal is proportional to the number of protons that generate it, and the proportionality constant in the ^1^H NMR spectrum is the same for all kinds of protons. This is true for all the signals appearing in the standard single-pulse spectrum and for the nonsuppressed ones of the NOESYGPPS spectrum. The equations employed for all these determinations are the same as in previous studies [17,33] and are provided in the Supplementary Materials Section.

Thus, for all the samples, the area of certain nonsuppressed signals of the multisuppressed NOESYGPPS pulse spectra was employed to estimate the concentration, expressed as µmol/mol of acyl groups and fatty acids (AG + FA), of several oxidation products: *Z*,*E*-conjugated dienic systems supported in chains also comprising a hydroperoxy (*Z*,*E*-CD-OOH) or a hydroxy group (*Z*,*E*-CD-OH); *Z*,*E*- or *E*,*Z*-conjugated dienes supported in chains also comprising a keto group (*Z*,*E*-CD=O); and several kinds of aldehydes (alkanals, *E*-2-alkenals, *E*,*Z*- and *E*,*E*-2,4-alkadienals and 4,5-epoxy-*E*-2-alkenals). The equations used for these estimations are provided as Supplementary Materials (see Section S2.1, Equations (S1)–(S4)).

The area of certain spectral signals in the standard pulse spectra was used to determine the molar proportions of the polyunsaturated fatty acyl chains present in certain samples, i.e., of linolenic (C18:3n-3) and linoleic (C18:2n-6) chains, using Equations (S5) and (S6) provided in Supplementary Materials (Section S2.2). The chemical shift assignments and multiplicities of the ^1^H NMR signals in CDCl_3_ of the main protons of fatty acyl chains are displayed in Table S2 of the Supplementary Materials Section. Given that pure ascorbyl palmitate (AP) and some of the studied supplements (VC2–4, VC6, VC7) included stearic and palmitic acid esters and phospholipids (see Table 1), the corresponding digests were not considered because a higher proportion of saturated or linoleic chains coming from the supplement itself would be present in them in comparison with linseed oil digest (DL). As this could distort the interpretation of the results obtained concerning the changes in the molar percentages of polyunsaturated chains after digestion, DL, DLAsc1–2, DLVC1 and DLVC5 were the only samples considered for discussion.

Moreover, the area of nonsuppressed signal **i** of the multisuppressed NOESYGPPS pulse spectra was employed to estimate gamma-tocopherol concentration in all the digest subjects of study, expressed as µmol/mol AG + FA, and using Equation (S7) provided in the Supplementary Materials (Section S2.3).

Finally, the area of certain spectral signals in the standard pulse spectrum was used to estimate the molar proportions of several kinds of glyceryl structures present in the lipid extracts of the digests, i.e., triglycerides (TG), 1,2- and 1,3-diglycerides (1,2-DG and 1,3-DG), 1- and 2-monoglycerides (1-MG and 2-MG) and glycerol (Gol). The chemical shift assignments and multiplicities of the ^1^H NMR signals in CDCl_3_ of the main protons of glycerides are displayed in Table S2 of the Supplementary Materials Section, and the equations employed were Equations (S8)–(S22) (Section S2.4). In addition, in order to estimate the extent of lipolysis in digestion from a physiological point of view, as the complete absorption of a TG only requires its conversion into one MG and two FA, the parameter lipid bioaccessibility (L_BA_%) was also estimated using Equation (S23). This parameter informs about the proportions of absorbable lipidic molecules released during digestion (MG and FA). As described before, taking into account that ascorbyl palmitate (AP) and some of the studied supplements (VC2–4, VC6, VC7) included stearic and palmitic acid esters and phospholipids (see Table 1) that would be hydrolyzed during digestion into fatty acids, DL, DLAsc1–2, DLVC1 and DLVC5 were the only samples considered for discussion.

### 2.5. Study through SPME-GC/MS of the Headspace Composition of the Digests

The study of several samples through Solid Phase Microextraction followed by Gas Chromatography/Mass Spectrometry (SPME-GC/MS) was carried out, following the same methodology and operating conditions as in previous works [17,34]. The samples, all analyzed in duplicate, were DL, DLVC1–7, DLAsc1–2 and DLAP, obtained in each digestion experiment carried out in triplicate (total *n* of 6). Likewise, after being submitted to digestion, digestive juices and supplements digested in the absence of oil were also studied as controls (data not shown). An amount of 0.5 g of the above-mentioned samples was transferred into a 10 mL screw-cap vial and the volatile components of the headspace were extracted by using a CombiPAL autosampler (Agilent Technologies, Santa Clara, CA, USA). The fiber used was coated with DVB/CAR/PDMS (Divinylbenzene/Carboxen/Polydimethylsiloxane, 50/30 μm film thickness, 1 cm long), acquired from Supelco (Merck KGaA), which was inserted into the headspace of the sample and maintained for 55 min at 50 °C, after a pre-equilibration time of 5 min. Afterward, the fiber containing the extracted components was desorbed for 10 min in the injection port (splitless mode with 5-min purge time) of a 7890 A gas chromatograph equipped with a 5975 C inert mass selective detector MSD with Triple Axis Detector (Agilent Technologies) and a computer operating with the ChemStation program. The column and the operation conditions employed were the same as in previous studies [17,34]. A reference sample of known composition was periodically analyzed in order to verify the extraction efficiency, SPME fiber repeatability and the performance of the equipment.

The identification of volatile components was mostly achieved by using commercial standards acquired from Merck KGaA. When standards were not available, matching of the mass spectra with those obtained from a commercial library at higher than 85% (Wiley W9N08, Mass Spectral Database of the National Institute of Standards and Technology (NIST)) was used as identification criterion. Mass spectra provided in scientific literature [35,36] were also taken into account for the identification of decatrienal and nonatrienal.

The semi quantification of identified volatile components was performed on the basis of the area counts of the base peak (Bp) of the mass spectrum of each compound divided by 10^6^. When the Bp of a compound overlapped with the same ion peak of the mass spectrum of another compound, an alternative ion peak was selected. Although the chromatographic response factor of each compound is different, the area counts thus determined are useful for the comparison of the abundance of each compound in the different samples. The detection limit was established at an abundance of 50,000 area counts.

### 2.6. Statistical Analysis

The significance of the differences on several determinations made among the samples was determined by one-way variance analysis (ANOVA) followed by Tukey b test at *p* < 0.05, using SPSS software v.26 (IBM, Endicott, NY, USA).

## 3. Results and Discussion

The effect of the presence of various Vitamin C-based supplements and of pure l-ascorbic acid and ascorbyl palmitate on certain important events occurring during the in vitro digestion of linseed oil is addressed. The advance of linseed oil oxidation is studied through the generation of oxidation compounds, the degradation of polyunsaturated fatty acyl chains and of gamma-tocopherol employing ^1^H NMR. The generation of volatile oxidation compounds during digestion is analyzed by SPME-GC/MS. Furthermore, the occurrence of lipolysis during digestion is estimated in some digests from ^1^H NMR data.

### 3.1. Effect of the Presence of Various Vitamin C-Based Supplements and of Pure l-Ascorbic Acid and Ascorbyl Palmitate on Linseed Oil Oxidation Occurring during Digestion

#### 3.1.1. Effect on the Formation of Oxidation Compounds That Can Be Detected through ^1^H NMR

Figure 1 shows certain regions of the multisuppressed ^1^H NMR spectra of linseed oil (L) together with those of the lipid extracts obtained after its in vitro digestion in the absence (DL) and in the presence of either ascorbic acid/salt (DLAsc1–2 and DLVC1–6) or ascorbyl palmitate (DLAP and DLVC7), in which proton signals of primary and secondary oxidation products can be observed. The assignment of these signals is provided in Table 2, and the estimated concentrations of the corresponding oxidation products detected in several lipidic samples, given in μmol/mol AG + FA, are shown in Table 3.

Figure 1 reveals that the oil subject of study (L) already showed a very slight degree of oxidation before being submitted to digestion. This is deduced from the presence in its multisuppressed ^1^H NMR spectrum of signals **a** and **h**, attributable to *Z**,**E*-conjugated dienes supported in octadecatrienoic fatty acyl chains also comprising a hydroxy group (*Z**,**E*-CD-OH) and to the aldehydic proton of alkanals, respectively. Their estimated concentrations (see Table 3) can be considered very low and similar to those found in other fresh edible oils rich in polyunsaturated acyl groups [17].

During the in vitro digestion of this highly polyunsaturated oil, not only the concentrations of these types of oxidation compounds increased (*p* > 0.05) slightly (see DL sample in Figure 1 and Table 3), but the generation of hydroperoxy-*Z*,*E*-conjugated dienes (*Z*,*E*-CD-OOH) derived from linolenic groups was also noticed (see signal **b** in DL spectrum), the latter being oxidation products formed in the highest proportion, in agreement with previous results [17,18].

The mixture of linseed oil with ascorbic acid, either pure (Asc1–2) or in the form of supplements containing basically this compound (VC1–4), provoked a clear enhancement of linseed oil oxidation during in vitro digestion. Thus, in the spectra corresponding to the lipids of the in vitro digests of the previously mentioned samples (DLAsc1–2 and DLVC1–4), increased intensities of the signals due to *Z,E*-CD-OH, *Z,E*-CD-OOH and alkanals (signals **a**, **b** and **h**, respectively) in comparison with DL spectrum were observed (see also concentrations in Table 3). Moreover, new signals attributable to protons of *Z*,*E*/*E*,*Z*-conjugated dienes of keto-octadecatrienoic structures were also detected (see signal **c** in Figure 1). In addition, the generation of several kinds of alpha,beta-unsaturated aldehydes was also favored, inferred from the appearance of doublets **d**, **e**, **f** and **g** due to the aldehydic proton of *E*-2-alkenals, *E*,*E*-2,4-alkadienals, 4,5-epoxy-*E*-2-alkenals and *E*,*Z*-2,4-alkadienals, respectively. Among them, the high reactivity of the oxygenated 4,5-epoxy-*E*-2-alkenals should be noticed [41]. These results are in line with those of Lee et al. [42], according to which Vitamin C (in the form of ascorbic acid) was very effective in decomposing linoleic acid-derived hydroperoxides to genotoxins, among which 4,5-epoxy-*E*-2-decenal was present.

Overall, as shown in Table 3, the estimated concentration of total conjugated dienic structures present in long chains in linseed oil digests was approximately 1.5–2-fold higher in DLAsc1–2 and DLVC1–4 than in DL, and that of total aldehydes from approximately 4-fold higher in DLVC2 up to ≈18-fold higher in DLAsc2. The ability of ascorbic acid to enhance lipid oxidation under certain conditions has been related to its capacity to reduce transition metal ions, which would in turn promote the formation of reactive free radicals that would favor lipid oxidation [21]. In this regard, it should be noticed that the presence of traces of metal ions in the reaction medium cannot be discarded, since previous studies have already reported their presence in edible vegetable oils, especially in the case of such virgin oils as the one employed in this study, as well as in porcine enzymes and extracts employed for preparing digestive juices [43,44]. Although the prooxidant behavior of ascorbic acid has been reported to be of relevance when this compound is present at low concentrations [45], the results of this study suggest that increased lipid oxidation also takes place in the presence of high concentrations of ascorbic acid in relation to that of lipids (170–2500 mg ascorbic acid/salt plus 0.5 g oil submitted to in vitro digestion).

It is also worth noticing that, whereas similar effects on conjugated dienes generation were observed for all doses of ascorbic acid, variations in the levels of aldehydes were found among digests, those coming from the mixtures of linseed oil with pure ascorbic acid (DLAsc1–2) showing the highest concentrations. However, no relationship was found between the amount of ascorbic acid and the level of oxidation products in the digests. As an example, despite a great difference between the amount of the ascorbic acid-based supplement VC1 added to the samples submitted to digestion (2500 mg) and that contained in VC4 (500 mg), the generation of oxidation products was of similar order in their corresponding digests (see DLVC1 and DLVC4 samples in Table 3).

The results obtained with the other two supplements based on ascorbic acid/salt, but also containing citric acid, carotenes and other components (VC5 and VC6), were completely different. It can be observed in DLVC5–6 spectra in Figure 1 that signal **b** of *Z*,*E*-CD-OOH is absent. In fact, the proton signals detected in DLVC5–6 spectra are the same ones detected in the starting oil spectrum and show similar intensities (see signals **a** and **h** in L spectrum). In contrast with that previously observed, the small advance of oxidation occurring during linseed oil digestion is avoided in the presence of VC5–6 supplements (see Figure 1). Quantitative data reported in Table 3 confirm these observations. As detailed in Table 1 of Section 2, VC5 and VC6 supplements are mixtures of ascorbic acid with citric acid and other components, such as carotenes, neohesperidin DC or orange juice concentrate, which could contribute to oxidation inhibition [46,47]. In this sense, citric acid is known to effectively delay lipid oxidation processes by chelating metal ions that could be involved in the mechanism of ascorbic acid to provoke oxidation. Moreover, Osborn-Barnes and Akoh [48] reported increased efficacy of citric acid in slowing down the generation of hydroperoxides and secondary oxidation products at acidic pH, such as that existing in the digestive tract.

It is noteworthy that similarly to that commented for DLVC5–6, in the linseed oil samples digested together with ascorbyl palmitate (DLAP, DLVC7), oxidation of linseed oil during digestion did not occur either, even though *E*-2-alkenals were generated in very small amounts (see Figure 1 and Table 3). These findings related to the effect of the presence of ascorbyl palmitate on the oxidation extent of linseed oil during digestion greatly contrast with those observed for ascorbic acid, both pure (DLAsc1–2) and in VC1–4 supplements (DLVC1–4). When trying to explain these differences, the polar paradox theory arises, even though factors other than polarity are known to influence prooxidant and antioxidant behaviors [49]. In contrast to ascorbic acid, which is highly soluble in water, the amphiphilic nature of ascorbyl palmitate would make these molecules locate at the interface of the lipid droplet, inside of which linseed oil glycerides would be found to be isolated from the aqueous media, where hydrophilic prooxidant species (such as metal ions or small reactive oxygen species) can be found. When ascorbyl palmitate is present at high concentrations, the contact between linseed oil polyunsaturated acyl chains and pro-oxidant species would be significantly hampered and would thus protect against oxidative degradation. Indeed, differences between ascorbic acid and ascorbyl palmitate regarding oxidation were observed in bulk oil by Frankel et al. [50], in such a way that ascorbic acid showed an antioxidant behavior, whilst ascorbyl palmitate acted as a prooxidant; however, this trend was inverted when studying oil/water emulsions, as occurs in this study.

#### 3.1.2. Effect on the Degradation of Polyunsaturated Acyl Groups during Digestion

As it is well known, fatty acyl chains presenting the highest number of double bonds are those showing the highest susceptibility toward oxidation. Thus, the higher the advance of oxidation, the higher degradation could be expected to affect polyunsaturated fatty acyl chains. It is worth reminding that, as indicated in Section 2.4.2, the only samples considered for studying the changes in the molar percentages of polyunsaturated chains as a result of digestion were DL, DLAsc1–2, DLVC1 and DLVC5. The molar percentages of linolenate and linoleate chains, before and after digestion, are reported in Table 4.

Linseed oil is especially rich in polyunsaturated acyl groups, the n-3 linolenate chains being the most abundant ones (≈52.0%). After in vitro digestion, the percentages of both linolenic and linoleic chains showed only slightly (*p* > 0.05) decreased values (≈51.3% and ≈17.5% in DL, respectively), indicating that they were degraded to a low extent during digestion. This is in accordance with previous studies on digestion of highly polyunsaturated oils [17,18,30,51].

When linseed oil is digested in the presence of ascorbic acid, clear differences can be observed among digests. Thus, Table 4 reveals that DLAsc1–2 and DLVC1 samples showed significantly (*p* < 0.05) lower molar proportion of linolenic AG + FA than DL (≈48% versus 51% in DL), whereas the molar percentage of linolenic AG + FA in DLVC5 was very similar to that of linseed oil before being subjected to in vitro digestion (L). These findings are in line with the outcomes of the study of the oxidation product profile of the above-referred samples (Section 3.1.1). This confirms that the presence of other components in addition to ascorbic acid in supplement VC5 seems to inhibit to a great extent the occurrence of oxidation during digestion of linseed oil.

#### 3.1.3. Effect on Gamma-Tocopherol Degradation during Digestion

The occurrence of oxidation during digestion can also lead to a loss of minor components naturally present in linseed oil to which antioxidant properties have been attributed, such as gamma-tocopherol. Taking this into account, the concentration of gamma-tocopherol was estimated in the oil before digestion and in the lipid extracts of the various in vitro digests, by measuring the area of singlet **i** at 6.36 ppm in the multisuppressed ^1^H NMR spectra (see Figure 1 and Table 2 for signal assignment).

As can be observed in Table 5, the concentration of gamma-tocopherol was greatly affected by the in vitro digestion process, exhibiting a value almost 3-fold lower in the digested lipid extract (DL) than in the starting oil (L). The decrease in the concentration of gamma-tocopherol naturally present in linseed and walnut oils during in vitro digestion was also observed in previous works [18,30,51].

When it comes to the digests corresponding to linseed oil digestion with only ascorbic acid (DLAsc1–2 and DLVC1–4), a total degradation of gamma-tocopherol took place during digestion (see the absence of singlet **i** in Figure 1). This could be expected, bearing in mind that these samples showed a higher oxidation level than DL. By contrast, in the presence of complex mixtures containing ascorbic acid/salt and citric acid, among other components, as is the case of VC5–6 supplements, degradation of gamma-tocopherol occurred to a very low extent, to the point that significant differences between the starting oil (L) and samples DLVC5–6 were not found (see Table 5 and signal **i** in Figure 1). Indeed, the mean concentration of gamma-tocopherol in DLVC5 and DLVC6 lipid extracts (413.0 and 406.4 μmol/mol AG + FA, respectively) was similar to that found in L (422.7 μmol/mol AG + FA). This indicates that, if oxidation reactions took place in these in vitro digests, their extent was not enough to cause a significant degradation of gamma-tocopherol. Similar results were obtained in the case of the samples digested with ascorbyl palmitate (DLAP and DLVC7). The slightly lower values (*p* > 0.05) for gamma-tocopherol concentration found in DLAP and DLVC7 (≈337 and 356 μmol/mol AG + FA) than in L could be explained to a certain extent by the increase in the total AG + FA in the digests due to the presence of the palmitate chains, rather than by the involvement of gamma-tocopherol in oxidation reactions.

In summary, the results concerning the analysis of gamma-tocopherol concentrations before and after in vitro digestion correspond with those related to the oxidation product profile of the various linseed oil digests (Section 3.1.1).

#### 3.1.4. Effect on the Formation of Volatile Oxidation Compounds Detected through SPME-GC/MS

The study and comparison of the profile of volatile components of several in vitro digests confirm the results obtained through ^1^H NMR on the different oxidation levels of the digests, regarding the generation of different kinds of oxidation compounds and the degradation of polyunsaturated chains and gamma-tocopherol. It must be highlighted that SPME-GC/MS can provide information on the specific nature of the individual volatile and semi-volatile lipid oxidation products present in the digests, which is important considering that some of them have been related to certain degenerative diseases [41].

Table 6 shows the abundance in the headspace of the different digests of selected volatile compounds known to be derived from linseed oil oxidation (aldehydes and some furan derivatives) from Maillard-type reactions (two alkyl-pyridines) and potentially from the degradation of ascorbic acid (furfural). It can be observed that the number and abundance of studied volatiles were far higher in DLAsc1–2 and DLVC1–4 than in DL, DLVC5–6, DLAP and DLVC7, these latter samples showing, in general, abundances of similar order. Thus, in line with the results provided through ^1^H NMR study, the oxidation level of linseed oil digests containing only ascorbic acid, added as pure compound (DLAsc1–2) or as supplement (DLVC1–4), was markedly higher than that reached by the rest.

Among the volatiles coming from linseed oil oxidation during digestion, it can be observed that alkadienals showed the highest abundances in digested samples, followed by alkanals and alkenals, which is in accordance with ^1^H NMR data (see Table 3). The main aldehydes detected were reactive 2,4-heptadienals [34,52,53]. Moreover, the oxygenated alpha,beta-unsaturated 4,5-epoxy-2-heptenals and 4,5-epoxy-2-decenals, whose potential toxicity has been described [41,52], were only found in DLAsc1–2 and DLVC1–4. As expected, considering linseed oil composition, the main furan derivatives detected in the digest headspaces were 2-pentyl-furan and 2-ethyl-furan [53]. The detection in DLAsc1–2 and DLVC1–4 of linolenic-derived alkenyl derivatives 2-(2-propenyl)- and 2-(2-pentenyl)-furan [54], and of two lactones 5-ethyl-2(5*H*)- and 5-pentyl-2(5*H*)-furanone, is noteworthy.

In addition to lipid oxidation, it is known that during in vitro digestion Maillard-type reactions can also take place. The highest abundances of volatiles coming from these reactions are expected to be present in the digests showing the highest oxidation level due to the higher amounts of carbonyl compounds derived from lipid oxidation. Indeed, in the headspace of DLAsc1–2 and DLVC1–4 samples, significantly increased concentrations of 3-ethyl-pyridine were found, together with 2-ethyl-pyridine, the latter not being detected in the rest of digests. Both compounds are known to be derived from reactions between 2,4-heptadienals and amino compounds [55].

Although Maillard-type reactions can also give rise to furfural, this volatile is considered to be the main product of ascorbic acid degradation [56,57]. Finholt et al. [57] even reported that under acidic and anaerobic conditions (such as those that could exist during the gastric step of the in vitro procedure), one furfural molecule appears to be formed from one ascorbic acid molecule. The extent of ascorbic acid degradation is expected to be in line with the extent of lipid oxidation. This is evidenced by the detection of significantly (*p* < 0.05) higher abundances of furfural in the headspace of DLVC1–3 samples in comparison with the others. It must be noted that the former are the digests of supplements that contain the highest amount of ascorbic acid (2500, 1500 and 1000 mg, respectively) and also show increased oxidation level.

### 3.2. Effect of the Presence of Some Vitamin C-Based Supplements and of Pure l-Ascorbic Acid on the Advance of Lipolysis during In Vitro Digestion of Linseed Oil

As expected, the enzymatic hydrolysis of linseed oil triglycerides (TG) took place through the in vitro digestion process, and several kinds of glycerides were generated by digestive lipases after breaking the ester bonds. As shown in Figure S1 of the Supplementary Materials Section, specific protons in TG, 1,2- and 1,3-diglycerides (1,2-DG, 1,3-DG), 2- and 1-monoglycerides (2-MG, 1-MG) and fatty acids (FA) generate certain non- or partially-overlapped signals in the standard spectra of the lipidic samples (see chemical shift assignments in Table S2) that, if present, can be detected and whose area can be employed for quantitative estimations. The molar proportions of several kinds of glyceryl structures present in the lipid extracts of certain in vitro digests (TG, 1,2-DG, 1,3-DG, 2-MG and 1-MG) and of glycerol (Gol), together with the Lipid bioaccessibility (L_BA_) parameter, are given in Table 7. As indicated in the Section 2, DL, DLAsc1–2, DLVC1 and DLVC5 were the only samples considered for the analysis of lipolysis extent.

The extent of hydrolysis during in vitro digestion of linseed oil was in line with previous studies on the same kind of oil [17,18,30]. As shown in Table 7, throughout the in vitro digestion process, approximately 24% of initial glycerides underwent complete hydrolysis into Gol, 48% of them were partially hydrolyzed (mainly into 2-MG and 1,2-DG), and 28% of triglycerides remained intact. Thus, in DL, L_BA_ was estimated to be approximately 59%. However, in the presence of Vitamin C-based supplements, the reached Gol % was significantly lower than in DL, indicating that complete hydrolysis of TG into Gol and FA was somehow hindered in the presence of ascorbic acid and under the conditions assayed. This effect was far more evident in the case of DLVC1, in which Gol barely accounted for 5%. In this latter sample, it is also worth noticing the significantly higher TG % that remained intact after in vitro digestion in comparison with DL. Both facts would explain the marked decrease in L_BA_ when linseed oil was co-digested with VC1 supplement. By contrast, no significant differences in L_BA_ parameter were observed among DL, DLAsc1–2 and DLVC5.

Therefore, the composition of Vitamin C-based supplements could also be relevant with regard to lipid bioaccessibility, since certain components might interfere with lipases activity. Thus, in the case of VC1 supplement, the high dose of ascorbic acid provided (2500 mg) could explain the lowered advance of lipolysis during digestion. In fact, Truong et al. [58] reported that ascorbic acid has a noticeable capacity to bind to bile acid, which would affect micelle formation and thus lipolysis, as well as certain ability of inhibiting pancreatic lipase. Nor can the potential covalent binding of ascorbic acid or its degradation products (i.e., furfural) to proteins [59] such as lipases through Maillard-type reactions be excluded, which could result in a decreased activity.

## 4. Conclusions

To the best of our knowledge, this is the first time that the effect of the presence of commercial supplements based on Vitamin C and of high concentrations of pure l-ascorbic acid and of ascorbyl palmitate on the advance of lipid oxidation during in vitro gastrointestinal digestion has been addressed. Under the conditions of this study, it has been evidenced that the chemical form through which Vitamin C is delivered (hydrophilic acid versus lipophilic derivative) and the presence of other ingredients, such as citric acid and carotenes in Vitamin C supplement formulations, play a key role in the extent of oxidation reactions under digestion conditions. In the presence of exclusively ascorbic acid, linseed oil oxidation was enhanced, leading to a significant increase in the formation of linolenic-derived conjugated hydroxy-dienes and of alkanals. Moreover, the generation of linolenic-derived keto-dienes and of several kinds of alpha,beta-unsaturated aldehydes, including potentially toxic 4,5-epoxy-2-alkenals that would be bioaccessible for absorption, was also noticed. In addition, gamma-tocopherol was degraded to a higher extent than in the absence of ascorbic acid, thus decreasing the nutritional value of oil. On the contrary, the small degree of oxidation taking place during linseed oil gastrointestinal digestion seemed to be inhibited in the presence of supplements containing either mixtures of ascorbic acid with other potentially antioxidant components, such as citric acid and carotenes, or in the presence of ascorbyl palmitate. In addition, Vitamin C supplementation could also have a negative impact on lipid bioaccessibilty, which was markedly decreased in the presence of the highest dose of l-ascorbic acid.

Considering the results obtained in this study, special attention should be paid when high concentrations of ascorbic acid are ingested daily for long periods of time, since they could favor the oxidation of small amounts of lipids simultaneously ingested, and even of biological lipids. In addition, they could also decrease the nutritional value of dietary lipids by enhancing the degradation of main and minor lipidic components of interest.

## Figures and Tables

**Table 1:** Composition of the different commercial supplements based on Vitamin C assayed (VC1–7) according to the label.

Supplement	Format	Composition
**VC1**	Powder	l-ascorbic acid powder (crystal form), whose recommended dose is a half tea spoon (2500 mg).
**VC2**	Capsule	l-ascorbic acid (1500 mg), rose hip powder (30 mg), bulking agents (microcrystalline cellulose, hydroxypropyl methylcellulose), anti-caking agents (magnesium stearate, silicon dioxide, stearic acid); coating agents (hydroxypropyl methylcellulose, glycerin); cellulose gum.
**VC3**	Capsule	l-ascorbic acid (1000 mg), anti-caking agent (magnesium stearate), hydroxypropyl methylcellulose capsule, coating agent (ethylcellulose).
**VC4**	Capsule	l-ascorbic acid (500 mg), bulking agents (microcrystalline cellulose, ethylcellulose), anti-caking agent (magnesium stearate), hydroxypropyl methylcellulose capsule.
**VC5**	Effervescent tablet	l-ascorbic acid (1000 mg), acidulant (citric acid), acidity regulator (sodium carbonate), sweetener (sorbitol), hardener (polyethylene glycol), colorant (carotenes), aroma-sweetener (sucralose, neohesperidin DC).
**VC6**	10 mL vial	Water, sodium ascorbate (1000 mg), orange juice concentrate, aromas, acidity regulator (citric acid), emulsifiers (phosphatidylcholine, polysorbate 20), preservative (potassium sorbate), thickening agent (gellan gum), sweetener (sucralose), colorant (beta-carotene).
**VC7**	Capsule	Ascorbyl palmitate (400 mg), cellulose capsule, bulking agent (cellulose).

**Table 2:** Chemical shift assignments and multiplicities of the ^1^H NMR signals in CDCl_3_ of the main protons of several primary and secondary oxidation products, as well as of gamma-tocopherol present in linseed oil and in the lipid extracts of the various digests. The signal letters correspond with those given in Figure 1.

Signal	Chemical Shift (ppm)	Multiplicity	Functional Group	
			Type of Protons	Compound
**Signals Related to Several Primary and Secondary Oxidation Products**				
**Conjugated Dienic Systems**				
**-**	5.38–5.46	m/dt	–C**H**=C**H**–C**H**=C**H**–	*Z,E*-conjugated double bonds associated with an hydroxy group (OH) in octadecatrienoic fatty acyl chain ^(I)^
**-**	5.69	dd		
**-**	5.99	t		
**a**	6.51	dd		
**-**	5.42	dt	–C**H**=C**H**–C**H**=C**H**–	*Z*,*E*-conjugated double bonds associated with an hydroperoxy group (OOH) in octadecatrienoic fatty acyl chain ^(II)^
**-**	5.56	dd		
**-**	6.00	t		
**b**	6.58	dd		
**c**	7.52/7.48	dd/t	–CO–CH=C**H**–CH=CH–	*Z*,*E*- and *E*,*Z*-conjugated double bonds associated with a keto group (C=O) in octadecatrienoic fatty acyl chain ^(III)^
**Aldehydes ^(IV)^**				
**d**	9.49	d	–C**H**O	*E*-2-alkenals
**e**	9.52	d	–C**H**O	*E*,*E*-2,4-alkadienals
**f**	9.55	d	–C**H**O	4,5-epoxy-*E*-2-alkenals
**g**	9.60	d	–C**H**O	*E*,*Z*-2,4-alkadienals
**h**	9.75	t	–C**H**O	alkanals
**Signals related to linseed oil minor components ^(V)^**				
**i**	6.36	s	–C_5_–**H**= 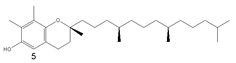	gamma-tocopherol

Abbreviations: d: doublet; t: triplet; m: multiplet; s: singlet. Assignments corresponding with: ^(I)^ [37]; ^(II)^ [38]; ^(III)^ [39]; ^(IV)^ [17,32]; ^(V)^ [40].

**Table 3:** Concentration of the various types of lipid oxidation products, estimated from ^1^H NMR data, in linseed oil before (L) and after in vitro digestion, either in the absence (DL) or in the presence of supplements and pure l-ascorbic acid and ascorbyl palmitate (DLVC1–7, DLAsc1–2, DLAP). Data are expressed as micromole per mole of acyl groups plus fatty acids (µmol/mol AG + FA). Data reported are mean values together with standard deviation from analysis in duplicate of three samples of each kind (*n* = 6). Different letters within each column indicate a significant difference (*p* < 0.05) among the samples.

	Conjugated dienes (µmol/mol AG + FA)				Aldehydes (µmol/mol AG + FA)					
	*Z*,*E*-CD-OOH	*Z*,*E*-CD-OH	*Z*,*E*-CD=O	Total CD	Alkanals	*E-*2-alk	*E*,*Z-*2,4-alk	*E*,*E-*2,4-alk	Epoxy-alk	Total Aldehydes
**L**	-	1178.7 ± 40.2 ^a^	-	1178.7 ± 40.2 ^a^	63.8 ± 3.7 ^a^	-	-	-	-	63.8 ± 3.7 ^a^
**DL**	1489.2 ± 269.4 ^a^	1438.7 ± 274.3 ^a^	-	2927.9 ± 462.8 ^b^	125.2 ± 29.2 ^ab^	-	-	-	-	125.2 ± 29.2 ^a^
**DLAsc1**	1913.0 ± 421.7 ^a^	2441.4 ± 59.1 ^c^	1759.4 ± 263.1 ^d^	6113.8 ± 721.2 ^c^	739.2 ± 19.2 ^e^	189.2 ± 21.5 ^ab^	179.6 ± 14.2 ^b^	626.6 ± 14.5 ^d^	115.5 ± 7.7 ^a^	1850.1 ± 49.0 ^d^
**DLAsc2**	1919.0 ± 619.5 ^a^	2457.3 ± 310.0 ^c^	1607.4 ± 231.2 ^cd^	5983.7 ± 952.6 ^c^	844.5 ± 81.5 ^f^	263.5 ± 57.1 ^b^	233.9 ± 10.2 ^c^	760 ± 43.4 ^e^	204.2 ± 34.6 ^b^	2306.5 ± 205.2 ^e^
**DLVC1**	1952.7 ± 278.8 ^a^	2478.3 ± 464.1 ^c^	1342.2 ± 147.0 ^bc^	5773.2 ± 887.9 ^c^	304.5 ± 24.4 ^c^	265.5 ± 43.9 ^b^	148.1 ± 65.7 ^b^	326.4 ± 49.0 ^b^	187.7 ± 41.8 ^b^	1232.2 ± 218.5 ^c^
**DLVC2**	2274.3 ± 474.0 ^a^	1623.3 ± 349.0 ^ab^	578.4 ± 106.3 ^a^	4476.0 ± 813.5 ^c^	182.8 ± 23.2 ^b^	109.4 ± 15.2 ^a^	67.9 ± 5.0 ^a^	93.3 ± 10.5 ^a^	67.8 ± 15.6 ^a^	521.2 ± 46.4 ^b^
**DLVC3**	1886.2 ± 449.4 ^a^	2292.4 ± 185.4 ^bc^	1104.4 ± 120.7 ^b^	5283.0 ± 755.4 ^c^	431.3 ± 27.0 ^d^	216.5 ± 36.1 ^ab^	159.9 ± 2.1 ^b^	307.7 ± 1.1 ^b^	169.1 ± 13.4 ^b^	1283.9 ± 48.7 ^c^
**DLVC4**	1653.4 ± 330.0 ^a^	2243.1 ± 193.9 ^bc^	1224.4 ± 342.3 ^bc^	5120.9 ± 474.9 ^c^	429.9 ± 15.3 ^d^	145.2 ± 36.5 ^a^	127.4 ± 25.0 ^b^	403.5 ± 69.3 ^c^	118.5 ± 28.4 ^a^	1287.5 ± 131.2 ^c^
**DLVC5**	-	1131.9 ± 215.8 ^a^	-	1131.9 ± 215.8 ^a^	162.6 ± 13.2 ^b^	-	-	-	-	162.6 ± 13.2 ^a^
**DLVC6**	-	1067.2 ± 171.1 ^a^	-	1067.2 ± 171.1 ^a^	175.4 ± 16.7 ^b^	-	-	-	-	175.4 ± 16.7 ^a^
**DLAP**	-	1225.0 ± 179.8 ^a^	-	1225.0 ± 179.8 ^a^	148.6 ± 26.3 ^ab^	185.7 ± 24.9 ^ab^	-	-	-	334.3 ± 2.0 ^ab^
**DLVC7**	-	1008.8 ± 224.8 ^a^	-	1008.8 ± 224.8 ^a^	138.3 ± 13.1 ^ab^	182.4 ± 86.8 ^ab^	-	-	-	320.6 ± 99.3 ^ab^

Abbreviations: *Z*,*E*-CD-OOH: *cis*,*trans*-conjugated dienic systems supported in chains also comprising a hydroperoxy group; *Z*,*E*-CD-OH: *cis*,*trans*-conjugated dienic systems supported in chains also comprising a hydroxy group; *Z*,*E*-CD=O: *cis*,*trans*- or *trans*,*cis*-conjugated dienic systems supported in chains also comprising a keto group. Total CD: sum of conjugated dienic systems supported in long chains also comprising a hydroperoxy, hydroxy or keto group. *E*-2-alk: *trans*-2-alkenals; *E*,*Z*-2,4-alk: *trans*,*cis*-2,4-alkadienals; *E*,*E*-2,4-alk: *trans*,*trans*-2,4-alkadienals; Epoxy-alk: 4,5-epoxy-*trans*-2-alkenals; -: not detected.

**Table 4:** Molar percentages estimated from ^1^H NMR data of linolenic and linoleic acyl groups and fatty acids referred to the total amount of acyl groups plus fatty acids (AG + FA), in linseed oil before (L) and after in vitro digestion, either in the absence (DL) or in the presence of certain supplements and pure l-ascorbic acid (DLVC1, DLVC5, DLAsc1–2). Data reported are mean values together with standard deviation from analysis in duplicate of three samples of each kind (*n* = 6). Different letters within each column indicate a significant difference (*p* < 0.05) among the samples.

	Polyunsaturated chains (Molar % of AG + FA)	
	Linolenic	Linoleic
**L**	52.0 ± 0.0 ^c^	18.9 ± 0.4 ^a^
**DL**	51.3 ± 0.3 ^bc^	17.5 ± 0.6 ^a^
**DLAsc1**	48.3 ± 0.5 ^a^	17.5 ± 1.6 ^a^
**DLAsc2**	48.2 ± 0.2 ^a^	16.6 ± 0.6 ^a^
**DLVC1**	48.7 ± 0.5 ^a^	17.8 ± 0.3 ^a^
**DLVC5**	51.5 ± 0.1 ^bc^	18.6 ± 1.3 ^a^

**Table 5:** Concentration of gamma-tocopherol estimated from ^1^H NMR data, expressed in μmol/mol AG + FA, in linseed oil before (L) and after in vitro digestion, either in the absence (DL) or in the presence of supplements and pure l-ascorbic acid and ascorbyl palmitate (DLVC1–7, DLAsc1–2, DLAP). Data reported are mean values together with standard deviation from analysis in duplicate of three samples of each kind (*n* = 6). Different letters indicate a significant difference (*p* < 0.05) among the samples.

	Gamma-Tocopherol (μmol/mol AG + FA)
**L**	422.7 ± 9.5 ^b^
**DL**	144.1 ± 100.2 ^a^
**DLAsc1**	-
**DLAsc2**	-
**DLVC1**	-
**DLVC2**	-
**DLVC3**	-
**DLVC4**	-
**DLVC5**	413.0 ± 26.0 ^b^
**DLVC6**	406.4 ± 95.4 ^b^
**DLAP**	336.5 ± 30.1 ^b^
**DLVC7**	355.9 ± 7.7 ^b^

-: not detected.

**Table 6:** Selected volatile compounds identified by means of SPME-GC/MS in the headspace of the in vitro digests of linseed oil in the absence (DL) and in the presence of supplements and pure l-ascorbic acid and ascorbyl palmitate (DLVC1–7, DLAsc1–2, DLAP). Data are expressed as area counts of the mass spectra base peak (Bp) of each compound divided by 10^6^. The abundances reported are mean values together with standard deviation from analysis in duplicate of three samples of each kind (*n* = 6). Different letters within each row indicate a significant difference (*p* < 0.05) among the samples.

Compound (Molecular Weight)	Bp	DL	DLAsc1	DLAsc2	DLVC1	DLVC2	DLVC3	DLVC4	DLVC5	DLVC6	DLAP	DLVC7
**Lipid oxidation-derived volatiles**												
**Alkanals**												
Pentanal (86) *	43	9.4 ± 1.3 ^a^	31.3 ± 6.8 ^bc^	34.4 ± 3.9 ^c^	32.9 ± 3.6 ^c^	30.0 ± 7.2 ^bc^	23.4 ± 7.3 ^bc^	21.1 ± 4.7 ^b^	2.5 ± 1.6 ^a^	1.2 ± 0.1 ^a^	7.0 ± 1.0 ^a^	10.5 ± 1.0 ^a^
Hexanal (100) *	44	158.5 ± 8.2 ^b^	395.0 ± 8.1 ^c^	415.2 ± 7.1 ^c^	503.7 ± 28.4 ^cd^	526.9 ± 181.2 ^d^	502.8 ± 44.9 ^cd^	391.2 ± 1.4 ^c^	46.3 ± 6.1 ^ab^	26.9 ± 1.6 ^a^	83.4 ± 26.4 ^ab^	133.4 ± 10.5 ^ab^
Heptanal (114) *	70	9.5 ± 1.0 ^a^	95.9 ± 2.5 ^b^	108.4 ± 3.0 b^bc^	134.0 ± 22.2 ^c^	95.5 ± 43.2 ^b^	92.7 ± 23.5 ^b^	98.1 ± 7.0 ^b^	7.6 ± 2.0 ^a^	10.1 ± 2.0 ^a^	14.4 ± 4.2 ^a^	19.6 ± 2.2 ^a^
Octanal (128) *	41	15.4 ± 0.3 ^a^	230.2 ± 22.5 ^d^	211.5 ± 35.8 ^cd^	184.1 ± 89.1 ^cd^	126.2 ± 61.5 ^bc^	146.8 ± 37.8 ^cd^	214.1 ± 40.3 ^cd^	48.6 ± 8.3 ^ab^	7.6 ± 2.8 ^a^	11.8 ± 2.9 ^a^	14.1 ± 0.8 ^a^
Nonanal (142) *	57	20.4 ± 0.5 ^a^	661.3 ± 20.1 ^e^	668.9 ± 66.6 ^e^	415.9 ± 64.0 ^cd^	276.5 ± 79.5 ^b^	330.7 ± 36.3 ^bc^	527.6 ± 105.8 ^d^	42.7 ± 2.4 ^a^	26.1 ± 3.6 ^a^	62.0 ± 12.6 ^a^	83.6 ± 15.3 ^a^
**Alkenals**												
*E-*2-Pentenal (84) is	55	2.5 ± 0.3 ^ab^	11.0 ± 0.9 ^bc^	15.5 ± 1.1 ^c^	25.1 ± 7.0 ^d^	30.5 ± 10.2 ^d^	14.1 ± 4.7 ^c^	11.4 ± 2.1 ^bc^	1.1 ± 0.3 ^a^	2.6 ± 0.2 ^ab^	4.6 ± 0.9 ^ab^	4.4 ± 0.1 ^ab^
*E*-2-Pentenal (84) *	55	9.8 ± 0.7 ^a^	294.7 ± 20.6 ^b^	339.1 ± 14.7 ^b^	352.7 ± 31.9 ^b^	334.7 ± 185.0 ^b^	278.3 ± 90.4 ^b^	309.0 ± 39.9 ^b^	4.2 ± 1.4 ^a^	4.4 ± 0.7 ^a^	4.5 ± 2.2 ^a^	7.5 ± 1.5 ^a^
*E*-2-Hexenal (98) *	41	4.4 ± 1.5 ^a^	179.4 ± 3.8 ^d^	137.1 ± 9.7 ^c^	98.3 ± 8.5 ^b^	80.5 ± 22.3 ^b^	85.7 ± 21.7 ^b^	148.1 ± 30.9 ^cd^	5.4 ± 0.5 ^a^	3.7 ± 0.3 ^a^	15.3 ± 7.3 ^a^	25.5 ± 4.3 ^a^
*Z-*4-Heptenal (112) *	41	1.5 ± 0.3 ^a^	23.4 ± 1.4 ^bc^	25.2 ± 1.8 ^c^	21.8 ± 0.1 ^bc^	16.9 ± 9.4 ^b^	19.6 ± 1.9 ^bc^	19.5 ± 3.0 ^bc^	-	0.4 ± 0.3 ^a^	1.4 ± 1.3 ^a^	3.0 ± 0.3 ^a^
*E-*2-Heptenal (112) *	41	14.9 ± 2.0 ^a^	186.9 ± 7.4 ^b^	230.0 ± 11.7 ^b^	349.2 ± 1.8 ^c^	229.0 ± 126.2 ^b^	223.8 ± 82.4 ^b^	180.5 ± 25.7 ^b^	4.9 ± 0.9 ^a^	2.2 ± 0.4 ^a^	1.9 ± 0.7 ^a^	2.2 ± 0.2 ^a^
*E-*2-Octenal (126) *	70	-	153.9 ± 37.1 ^b^	264.3 ± 57.6 ^c^	213.8 ± 52.0 ^bc^	143.4 ± 58.1 ^b^	181.7 ± 59.2 ^bc^	199.9 ± 41.7 ^bc^	-	4.3 ± 0.6 ^a^	-	6.9 ± 1.8 ^a^
*E-*2-Nonenal (140) *	55	1.9 ± 0.1 ^a^	110.1 ± 23.3 ^bc^	134.6 ± 46.5 ^c^	59.8 ± 16.9 ^ab^	21.2 ± 10.2 ^a^	39.7 ± 10.7 ^a^	118.0 ± 43.2 ^c^	8.5 ± 0.8 ^a^	6.2 ± 1.8 ^a^	3.7 ± 1.4 ^a^	3.7 ± 1.6 ^a^
*E-*2-Decenal (154) *		-	95.5 ± 5.8 ^b^	159.1 ± 7.0 ^c^	140.2 ± 27.2 ^c^	65.5 ± 18.3 ^b^	101.0 ± 32.4 ^b^	97.4 ± 14.2 ^b^	-	-	-	-
*E-*2-Undecenal (168) *		-	53.6 ± 3.3 ^d^	59.6 ± 9.6 ^d^	39.9 ± 25.1 ^cd^	10.9 ± 4.4 ^ab^	28.2 ± 8.5 ^bc^	44.4 ± 15.5 ^cd^	1.5 ± 0.4 ^a^	-	-	-
**Alkadienals and Alkatrienals**												
2,4-Pentadienal (82)	82	0.7 ± 0.3 ^a^	5.0 ± 0.5 ^b^	7.2 ± 0.8 ^c^	4.2 ± 0.8 ^b^	4.3 ± 2.9 ^b^	3.3 ± 0.9 ^b^	3.4 ± 1.1 ^b^	-	-	-	-
2,4-Hexadienal (96)	81	10.1 ± 0.2 ^a^	119.6 ± 5.4 ^b^	199.0 ± 16.3 ^c^	199.2 ± 7.8 ^c^	99.9 ± 62.2 ^b^	140.7 ± 53.1 ^bc^	139.3 ± 22.5 ^bc^	4.7 ± 0.9 ^a^	2.7 ± 0.3 ^a^	4.6 ± 1.0 ^a^	8.5 ± 1.2 ^a^
*E*,*Z-*2,4-Heptadienal (110) *	81	19.3 ± 2.2 ^a^	732.7 ± 38.8 ^b^	780.4 ± 1.9 ^b^	800.8 ± 85.4 ^b^	611.2 ± 181.7 ^b^	756.4 ± 142.6 ^b^	775.0 ± 64.8 ^b^	12.8 ± 2.5 ^a^	13.9 ± 3.0 ^a^	4.2 ± 1.1 ^a^	6.3 ± 0.9 ^a^
*E*,*E-*2,4-Heptadienal (110) *	81	29.1 ± 2.9 ^a^	1320.6 ± 155.7 ^c^	1312.2 ± 102.5 ^c^	1286.9 ± 173.6 ^c^	927.8 ± 323.6 ^b^	1525.0 ± 146.5 ^c^	1506.5 ± 148.5 ^c^	27.9 ± 1.0 ^a^	31.5 ± 4.3 ^a^	7.3 ± 1.5 ^a^	10.1 ± 2.4 ^a^
*E*,*E-*2,4-Octadienal (124) *	81	-	19.7 ± 2.2 ^b^	17.7 ± 2.6 ^b^	34.1 ± 5.2 ^c^	16.5 ± 4.9 ^b^	21.9 ± 4.9 ^b^	23.5 ± 7.9 ^b^	-	0.8 ± 0.1 ^a^	-	-
2,6-Nonadienal (138)	41	-	483.9 ± 39.8 ^c^	470.0 ± 81.0 ^c^	173.2 ± 75.8 ^b^	34.2 ± 14.9 ^a^	108.1 ± 1.3 ^ab^	465.5 ± 91.0 ^c^	26.9 ± 5.4 ^a^	14.8 ± 5.3 ^a^	26.4 ± 10.3 ^a^	25.5 ± 12.0 ^a^
3,6-Nonadienal (138)	67	-	18.9 ± 3.6 ^bc^	37.3 ± 8.1 ^d^	22.5 ± 2.2 ^bc^	14.5 ± 5.6 ^bc^	13.0 ± 0.4 ^b^	30.5 ± 12.1 ^cd^	-	-	1.3 ± 0.3 ^a^	0.9 ± 0.3 ^a^
*E*,*E-*2,4-Nonadienal (138)	81	1.1 ± 0.1 ^a^	79.4 ± 3.6 ^bc^	144.8 ± 15.2 ^d^	92.1 ± 9.9 ^c^	22.8 ± 9.4 ^b^	54.6 ± 24.2 ^b^	56.4 ± 19.0 ^b^	-	-	0.7 ± 0.4 ^a^	0.9 ± 0.3 ^a^
*E*,*Z-*2,4-Decadienal (152) *	81	-	78.2 ± 4.7 ^c^	69.0 ± 8.1 ^c^	41.4 ± 17.6 ^b^	13.3 ± 5.6 ^a^	43.7 ± 11.8 ^b^	77.9 ± 18.6 ^c^	-	-	-	-
*E*,*E-*2,4-Decadienal (152) *	81	0.5 ± 0.1 ^a^	180.4 ± 14.1 ^d^	151.7 ± 10.8 ^cd^	53.7 ± 19.9 ^b^	12.5 ± 3.3 ^a^	63.6 ± 11.4 ^b^	131.9 ± 41.0 ^c^	1.6 ± 0.1 ^a^	3.6 ± 0.6 ^a^	0.2 ± 0.0 ^a^	0.4 ± 0.1 ^a^
2,4,6-Nonatrienal (136)	79	0.6 ± 0.1 ^a^	37.4 ± 3.4 ^c^	72.1 ± 12.7 ^d^	32.3 ± 0.0 ^c^	6.1 ± 1.8 ^ab^	14.6 ± 5.3 ^b^	16.1 ± 2.2 ^b^	-	-	0.3 ± 0.3 ^a^	0.4 ± 0.4 ^a^
2,4,6-Nonatrienal (136) is	79	-	20.8 ± 0.1 ^b^	34.7 ± 5.5 ^c^	19.8 ± 3.2 ^b^	4.5 ± 1.1 ^a^	11.3 ± 4.4 ^ab^	23.0 ± 13.3 ^bc^	-	-	-	-
2,4,7-Decatrienal (152)	79	-	13.2 ± 0.8 ^cd^	15.1 ± 2.2 ^d^	9.7 ± 2.0 ^bc^	3.6 ± 1.5 ^a^	7.7 ± 2.2 ^b^	16.5 ± 3.9 ^d^	-	-	-	-
***Oxygenated aldehydes***												
4,5-Epoxy-2-heptenal (126) *	68	-	204.6 ± 52.6 ^bc^	352.5 ± 32.4 ^d^	296.2 ± 12.4 ^cd^	161.6 ± 105.5 ^b^	157.9 ± 102.4 ^b^	101.7 ± 7.5 ^ab^	-	-	-	-
4,5-Epoxy-2-heptenal (126) is	68	-	63.4 ± 16.7 ^b^	152.2 ± 24.9 ^c^	115.6 ± 8.5 ^c^	58.5 ± 39.2 ^b^	59.5 ± 34.9 ^b^	34.3 ± 2.6 ^ab^	-	-	-	-
4,5-Epoxy-2-decenal (168) *	68	-	10.7 ± 0.6 ^c^	19.5 ± 0.8 ^d^	10.3 ± 6.6 ^c^	5.6 ± 3.9 ^bc^	5.8 ± 2.3 ^bc^	4.7 ± 0.2 ^ab^	-	-	-	-
4,5-Epoxy-2-decenal (168) is	68	-	30.4 ± 0.1 ^c^	51.8 ± 3.0 ^d^	27.8 ± 17.4 ^bc^	15.0 ± 11.0 ^ab^	14.8 ± 7.0 ^ab^	10.4 ± 0.7 ^a^	-	-	-	-
***Furan derivatives***												
Furan, 2-ethyl- (96) *	81	6.5 ± 1.0 ^a^	51.8 ± 4.3 ^b^	51.0 ± 3.2 ^b^	51.4 ± 11.2 ^b^	44.9 ± 18.1 ^b^	42.5 ± 10.7 ^b^	45.1 ± 14.7 ^b^	1.5 ± 0.4 ^a^	0.6 ± 0.0 ^a^	2.7 ± 0.5 ^a^	4.1 ± 0.1 ^a^
Furan, 2-pentyl- (138) *	81	24.0 ± 2.6 ^a^	303.9 ± 14.0 ^e^	344.4 ± 15.8 ^e^	98.9 ± 26.4 ^bc^	57.4 ± 14.0 ^ab^	117.8 ± 21.4 ^c^	235.1 ± 50.1 ^d^	22.7 ± 3.5 ^a^	19.7 ± 1.5 ^a^	22.0 ± 3.3 ^a^	33.8 ± 1.3 ^a^
Furan, 2-(2-propenyl)- (108)	108	-	11.6 ± 0.6 ^bc^	11.2 ± 0.3 ^bc^	13.7 ± 0.1 ^c^	6.5 ± 3.9 ^a^	10.4 ± 2.6 ^bc^	9.9 ± 1.5 ^b^	-	-	-	-
Furan, 2-(2-pentenyl)- (136)	107	-	25.9 ± 2.3 ^c^	34.3 ± 6.9 ^c^	5.8 ± 0.5 ^a^	3.1 ± 0.9 ^a^	3.7 ± 1.0 ^a^	15.1 ± 9.5 ^b^	-	-	-	-
2(5H)-Furanone, 5-ethyl- (112)	83	-	9.1 ± 1.8 ^b^	13.9 ± 2.6 ^b^	19.2 ± 5.1 ^b^	17.2 ± 10.2 ^b^	17.6 ± 6.7 ^b^	12.5 ± 0.4 ^b^	-	nq	-	-
2(5H)-Furanone,5- pentyl- (154)	84	-	3.7 ± 0.5 ^a^	5.1 ± 0.5 ^a^	4.2 ± 1.5 ^a^	3.2 ± 1.2 ^a^	4.0 ± 1.4 ^a^	3.2 ± 0.8 ^a^	-	-	-	-
**Maillard-type reactions and Ascorbic acid degradation-derived volatiles**												
Pyridine, 2-ethyl- (107) *	106	-	17.0 ± 0.9 ^e^	7.5 ± 0.2 ^c^	4.9 ± 0.9 ^b^	1.7 ± 0.9 ^a^	8.1 ± 1.5 ^c^	11.1 ± 2.3 ^d^	-	-	-	-
Pyridine, 3-ethyl- (107) *	92	6.9 ± 0.4 ^a^	33.7 ± 1.4 ^d^	23.8 ± 1.4 ^c^	22.5 ± 3.3 ^c^	14.0 ± 6.8 ^b^	26.8 ± 5.5 ^c^	23.3 ± 0.6 ^c^	2.8 ± 0.8 ^a^	1.1 ± 0.6 ^a^	5.6 ± 0.8 ^a^	6.2 ± 0.7 ^a^
Furfural (96)	96	20.0 ± 1.6 ^ab^	19.4 ± 0.3 ^ab^	23.2 ± 4.1 ^ab^	72.0 ± 8.6 ^e^	55.4 ± 2.0 ^d^	38.4 ± 0.2 ^c^	27.6 ± 0.4 ^b^	26.7 ± 2.6 ^b^	13.4 ± 1.4 ^a^	13.8 ± 4.1 ^a^	23.5 ± 3.7 ^ab^

* Asterisked compounds were acquired commercially and used as standards for identification purposes. Abbreviations: -: not detected; is: isomer; nq: not quantifiable.

**Table 7:** Lipid bioaccessibility (L_BA_) and molar percentages estimated from ^1^H NMR data of the different kinds of glycerides present in linseed oil before (L) and after in vitro digestion, either in the absence (DL) or in the presence of certain supplements and pure l-ascorbic acid (DLVC1, DLVC5, DLAsc1–2). Data reported are mean values together with standard deviation from analysis in duplicate of three samples of each kind. Different letters within each column indicate a significant difference (*p* < 0.05) among the samples.

	L_BA_ (%)	TG %	1,2-DG %	1,3-DG %	2-MG %	1-MG %	Gol %
**L**	0.7 ± 0.1 ^a^	98.3 ± 0.1 ^d^	1.5 ± 0.0 ^a^	-	-	-	0.2 ± 0.1 ^a^
**DL**	59.4 ± 6.5 ^c^	28.0 ± 5.2 ^a^	15.8 ± 1.8 ^b^	3.0 ± 0.1 ^b^	25.7 ± 3.2 ^de^	3.6 ± 1.2 ^bc^	23.8 ± 2.8 ^c^
**DLAsc1**	60.1 ± 6.6 ^c^	22.5 ± 5.2 ^a^	23.2 ± 2.5 ^c^	3.0 ± 0.8 ^b^	30.7 ± 6.5 ^e^	4.1 ± 1.1 ^c^	16.6 ± 4.0 ^b^
**DLAsc2**	56.9 ± 1.1 ^c^	19.2 ± 2.2 ^a^	34.1 ± 1.7 ^d^	1.7 ± 0.1 ^b^	29.5 ± 1.2 ^de^	1.1 ± 0.3 ^a^	14.3 ± 1.0 ^b^
**DLVC1**	27.7 ± 1.6 ^b^	56.3 ± 1.7 ^c^	20.8 ± 3.2 ^bc^	3.2 ± 0.9 ^b^	12.8 ± 1.1 ^b^	1.6 ± 1.0 ^ab^	5.4 ± 2.4 ^a^
**DLVC5**	51.0 ± 2.5 ^c^	27.1 ± 1.9 ^a^	30.4 ± 0.8 ^d^	2.3 ± 0.5 ^b^	22.6 ± 0.1 ^cd^	2.3 ± 0.7 ^abc^	15.2 ± 2.9 ^b^

Abbreviations: TG: triglycerides; DG: diglycerides; MG: monoglycerides; Gol: glycerol; -: not detected.

## Data Availability

The datasets generated for this study are available on request to the corresponding author.

## Supplementary Materials

The following are available online at https://www.mdpi.com/article/10.3390/foods11010058/s1; Figure S1: ^1^H NMR standard pulse spectrum of linseed oil (L) and that of the lipid extract of the corresponding in vitro digest (DL). Spectral region between 3.5 and 5.2 ppm is properly enlarged in order to better observe signals generated by protons in partial glycerides. Signal letters correspond with those in Table S2; Table S1: Concentration (units given in brackets) of each one of the components present in the juices used in the in vitro digestion procedure, together with the pH of the latter; Table S2: Chemical shift assignments and multiplicities of the ^1^H NMR signals in CDCl_3_ of the main protons of glycerides and fatty acids present in the lipid samples subject of study.
