# Supplementary material for: Different Effects of Vitamin C-Based Supplements on the Advance of Linseed Oil Component Oxidation and Lipolysis during In Vitro Gastrointestinal Digestion
Source: Foods. 2021 Dec 27;11(1):58. doi: 10.3390/foods11010058 (PMC8750871; doi:10.3390/foods11010058)
Supplement: Supplementary file 1 [file foods-11-00058-s001.zip › foods-1501182-supplementary.pdf]

foods

SUPPLEMENTARY MATERIAL

Different Effects of Vitamin C-Based Supplements on the Advance of Linseed Oil Component Oxidation and Lipolysis during In Vitro Gastrointestinal Digestion

Bárbara Nieva-Echevarría, Encarnación Goicoechea, Patricia Sopelana and María D. Guillén *

Food Technology. Faculty of Pharmacy. Lascaray Research Center. University of the Basque Country (UPV/EHU). 01006 Vitoria-Gasteiz, Spain; barbara.nieva@ehu.eus; encarnacion.goicoechea@ehu.eus; patricia.sopelana@ehu.eus

* Correspondence: mariadolores.guillen@ehu.eus; Tel.: +34 945013081

Citation: Nieva-Echevarría, B.; Goicoechea, E.; Sopelana, P.; Guillén, M.D. Different Effects of Vitamin C-Based Supplements on the Advance of Linseed Oil Component Oxidation and Lipolysis during In Vitro Gastrointestinal Digestion. *Foods* **2022**, *11*, 58. https://doi.org/10.3390/foods11010058

Academic Editor: Helena Teixeira Godoy

Received: 24 November 2021
Accepted: 22 December 2021
Published: 27 December 2021

Publisher's Note: MDPI stays neutral with regard to jurisdictional claims in published maps and institutional affiliations.

Copyright: © 2021 by the authors. Licensee MDPI, Basel, Switzerland. This article is an open access article distributed under the terms and conditions of the Creative Commons Attribution (CC BY) license (https://creativecommons.org/licenses/by/4.0/).

1. *Description of the in vitro gastrointestinal digestion procedure*

The digestion model employed in this study is a semi-static *in vitro* procedure consisting in three consecutive steps that simulate digestive processes occurring in mouth, stomach and duodenum. It was developed for fed state by Versantvoort et al. (2004, 2005) in the National Institute for Public Health and the Environment of The Netherlands, and slightly modified later in order to obtain a lipolysis degree similar to that reached *in vivo* (Nieva-Echevarría et al. 2016). Thus, simulated digestive juices (saliva, gastric juice, duodenal juice and bile) were prepared in accordance with the original model but included the following modifications: lipase from *Aspergillus niger* (100 U/mL) was added to the gastric juice and a lower concentration of bovine bile (18.75 instead of 30.00 g/L) was used for preparing bile juice. The composition of digestive juices is shown in **Table S1**. All the reagents and enzymes were acquired from Sigma-Aldrich (St. Louis, MO, USA).

Digestive juices were freshly made and, just before the *in vitro* digestion experiments, heated to 37±2 ºC. The digestion experiment began by mixing 6 mL of saliva with the sample (0.5 g of linseed oil, either alone or together with vitamin C supplement) in a Corning Falcon-50 mL tube. After 5 min of incubation, 12 mL of simulated gastric juice were added into the tube and the mixture was subjected to rotation head-over-heels at 40 rpm for 2 h inside an IF450 incubator (Memmert GmbH + Co. KG, Schwabach, Germany) set at 37±2 ºC. One hour after starting the gastric digestion, the pH of the mixture was measured and set at 2.5±0.5 with HCl (37%) to mimic the gradual acidification of the chyme occurring *in vivo*. After 2 h of gastric digestion, 2 mL of sodium bicarbonate solution (1 M), 12 mL of duodenal juice and 6 mL of bile were added. The pH of the mixture was set at 6.5±0.5 with NaOH (1 M) and the tube was rotated again at 40 rpm for 4 h inside the incubator at 37±2 ºC.

Table S1. Concentration (units given in brackets) of each one of the components present in the juices used in the *in vitro* digestion procedure, together with the pH of these latter.

Components	Saliva	Gastric juice	Duodenal juice	Bile juice
KCl (mmol/L)	12.02	11.06	7.57	5.05
NaCl (mmol/L)	5.10	47.09	119.98	89.99
$NaHCO_3$ (mmol/L)	20.17	-	40.33	68.86
NaH_2PO_4 (mmol/L)	7.40	0.22	-	-
NH_4Cl (mmol/L)	-	5.72	-	-
KH_2PO_4 (mmol/L)	-	-	0.59	-
Na_2SO_4 (mmol/L)	4.79	-	-	-
KSCN (mmol/L)	2.06	-	-	-
$MgCl_2$ (mmol/L)	-	-	0.53	-
$CaCl_2$*$2H_2O$ (mmol/L)	-	2.72	1.36	1.51
HCl (37%) (mL/L)	-	6.50	0.18	0.15
Urea (mmol/L)	3.33	1.42	1.67	4.16
Glucose (mmol/L)	-	3.61	-	-
Glucuronic acid (mmol/L)	-	0.10	-	-
Uric acid (mmol/L)	0.09	-	-	-
Glucoseamine hydrochloride (mmol/L)	-	1.53	-	-
Bovine serum albumin (g/L)	-	1.00	1.00	1.80
Mucin (g/L)	0.025	3.00	-	-
Aspergillus oryzae α-amylase (g/L)	0.29	-	-	-
Aspergillus niger lipase (U/mL)	-	100	-	-
Pepsin (g/L)	-	2.50	-	-
Pancreatin (g/L)	-	-	9.00	-
Lipase type II from porcine pancreas (g/L)	-	-	1.50	-
Bovine bile extract (g/L)	-	-	-	18.75
**pH**	6.8±0.2	1.6±0.3	8.1±0.2	8.2±0.2

2. *Study by means of ^{1}H NMR of linseed oil and digested lipid extracts*

2.1 Quantitative determination of the several primary and secondary oxidation products

The concentration of the several oxidation products (*cis,trans*-conjugated dienic systems supported in chains having also an hydroperoxy group, *Z,E*-CD-OOH; *cis,trans*-conjugated dienic systems supported in chains having also an hydroxy group, *Z,E-CD*-OH; *cis,trans*- or *trans,cis*-conjugated dienes supported in chains having also a keto group, *Z,E*-CD=O; and different kinds of aldehydes), expressed as micromol per mol of fatty acyl chains (μmol/molAG+FA), was estimated by using the following equations:

***Z,E*-CD-OOH** (μmol/molAG+FA) = $2*10^6*A_b/A_F$ [eq.S1]
***Z,E*-CD-OH** (μmol/molAG+FA) = $2*10^6*A_a/A_F$ [eq.S2]
***Z,E*-CD=O** (μmol/molAG+FA) = $2*10^6*A_c/A_F$ [eq.S3]
Aldehydes (μmol/molAG+FA) = $2*10^6*A_{Ald}/A_F$ [eq.S4]

where A_F is the area of signal **F** due to the protons in *alpha*-position in relation to the carbonyl group of AG plus to the carboxyl group of FA (see **Figure S1** and **Table S2**), modified or not, as well as to carbonyl groups of oxidation products like aldehydes or

ketones. However, as the concentration of these latter is negligible in relation with that of fatty acyl chains, the inclusion in this signal of methylenic protons in *alpha* position in relation to carbonyl groups different from that of fatty acyl chains is considered to not affect the quantitative determinations in which area of signal **F** is involved. A_a, A_b, and A_c are the area of signal **a**, signal **b,** and signal **c** centered at approximately 6.51 ppm, 6.58 ppm and 7.52/7.48 ppm respectively; and, A_{Ald} is the area of the signal of the aldehydic proton of each one of the aldehydes detected (see assignments in **Table 2**).

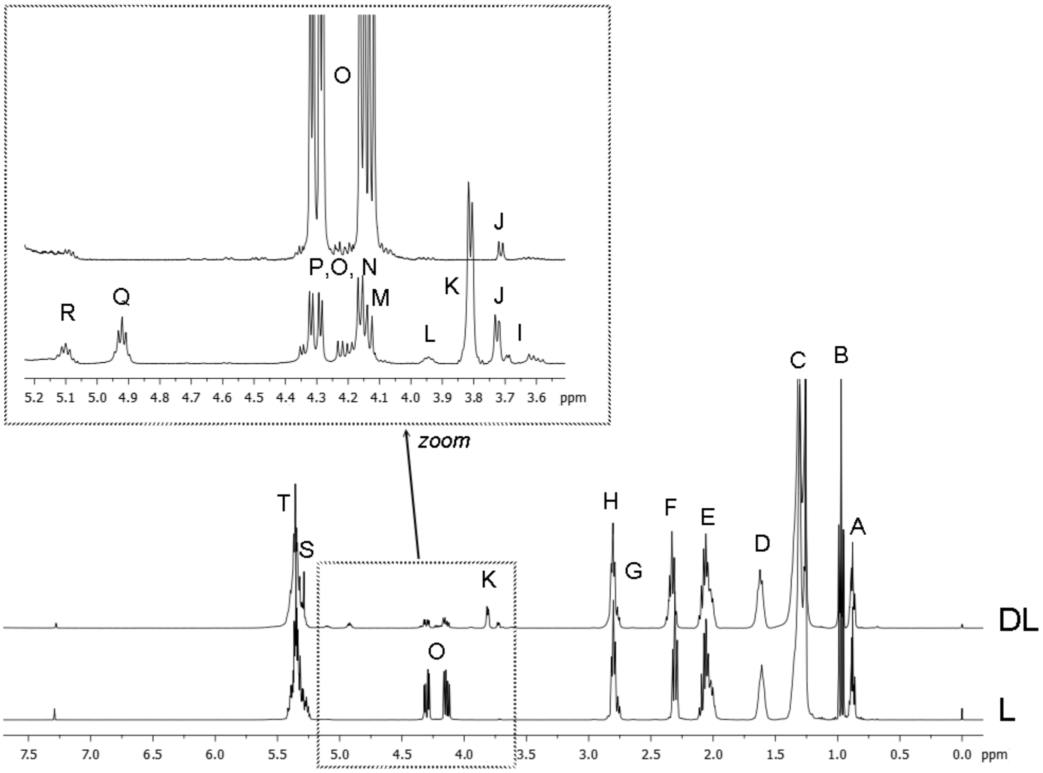

Figure S1. ^{1}H NMR standard pulse spectrum of linseed oil (L) and that of the lipid extract of the corresponding in vitro digest (DL). Spectral region between 3.5 and 5.2 is properly enlarged in order to better observe signals generated by protons in partial glycerides. Signal letters agree with those in Table S2.

2.2 Quantitative determination of the several kinds of polyunsaturated acyl groups and fatty acids

The estimation on the molar percentage of linolenic (**Ln%**) and linoleic (**L%**) fatty acyl chains in relation to the total number of moles of acyl groups and fatty acids present in the lipid samples can be done by means of the following equations:

Ln% $= 100{*}A_H/(2{*}A_F)$ [eq.S5]

L% $= 100{*}A_G/A_F$ [eq.S6]

where A_F, A_H and A_G are the areas of signals **F**, **H** and **G** indicated in **Table S2** and **Figure S1**. It must be noted that, because of the partial overlapping of signals **H** and **G**, the areas A_H and A_G were previously corrected by using standard compounds.

2.3 Quantitative determination of gamma-tocopherol

The molar concentration of *gamma*-tocopherol in linseed oil samples before and after *in vitro* digestion was estimated as follows:

***gamma*-tocopherol** (μmol/molAG+FA) = $2*10^{6}*A_i/A_F$ [eq.S7]

where $\mathbf{A_i}$ is the area of singlet at 6.36 ppm corresponding to the proton bounded to carbon atom 5 of *gamma*-tocopherol (see assignment in **Table 2**).

Table S2. Chemical shift assignments and multiplicities of the ^{1}H NMR signals in $CDCl_3$ of the main protons of glycerides and fatty acids present in the lipid samples subject of study (Guillén & Ruiz; 2004, 2005; Nieva-Echevarría et al. 2014).

Signal	Chemical shift (ppm)	Multi-plicity	Functional group	
			Type of protons	Compound
			**Main acyl groups (AG) and fatty acids (FA)** [(a)]	
**A**	0.88	t	$-C\underline{\mathbf{H}}_3$	saturated, monounsaturated *omega*-**9 AG** and **FA**
	0.89	t	$-C\underline{\mathbf{H}}_3$	linoleic **AG** and **FA**
**B**	0.97	t	$-C\underline{\mathbf{H}}_3$	linolenic **AG** and **FA**
**C**	1.19-1.42	m[§]	$-(C\underline{\mathbf{H}}_2)_n-$	**AG** and **FA**
**D**	1.61	m	$-OCO-CH_2-C\underline{\mathbf{H}}_2-$	**AG** in **TG**
	1.62	m	$-OCO-CH_2-C\underline{\mathbf{H}}_2-$	**AG** in **1,2-DG**
	1.63	m	$-OCO-CH_2-C\underline{\mathbf{H}}_2-$, $COOH-CH_2-C\underline{\mathbf{H}}_2-$	**AG** in **1,3-DG**, **1-MG** and **FA**
	1.64	m	$-OCO-CH_2-C\underline{\mathbf{H}}_2-$	**AG** in **2-MG**
**E**	1.92-2.15	m[§§]	$-C\underline{\mathbf{H}}_2-CH=CH$	**AG** and **FA**
**F**	2.26-2.36	dt	$-OCO-C\underline{\mathbf{H}}_2-$	**AG** in **TG**
	2.33	m	$-OCO-C\underline{\mathbf{H}}_2-$	**AG** in **1,2-DG**
	2.35	t	$-OCO-C\underline{\mathbf{H}}_2-$, $COOH-C\underline{\mathbf{H}}_2-$	**AG** in **1,3-DG**, **1-MG** and **FA**
	2.38	t	$-OCO-C\underline{\mathbf{H}}_2-$	**AG** in **2-MG**
**G**	2.77	t	$=HC-C\underline{\mathbf{H}}_2-CH=$	linoleic **AG** and **FA**
**H**	2.80	t	$=HC-C\underline{\mathbf{H}}_2-CH=$	linolenic **AG** and **FA**
**I**	3.65	ddd	$ROCH_2-CHOH-C\underline{\mathbf{H}}_2OH$	glyceryl group in **1-MG**
**J**	3.73	m[§§§]	$ROCH_2-CH(OR')-C\underline{\mathbf{H}}_2OH$	glyceryl group in **1,2-DG**
**K**	3.84	m[§§§]	$HOC\underline{\mathbf{H}}_2-CH(OR)-C\underline{\mathbf{H}}_2OH$	glyceryl group in **2-MG**
**L**	3.94	m	$ROCH_2-C\underline{\mathbf{H}}OH-CH_2OH$	glyceryl group in **1-MG**
**M**	4.05-4.21	m	$ROC\underline{\mathbf{H}}_2-C\underline{\mathbf{H}}OH-C\underline{\mathbf{H}}_2OR'$	glyceryl group in **1,3-DG**
**N**	4.18	ddd	$ROC\underline{\mathbf{H}}_2-CHOH-CH_2OH$	glyceryl group in **1-MG**
**O**	4.22	dd,dd	$ROC\underline{\mathbf{H}}_2-CH(OR')-C\underline{\mathbf{H}}_2OR''$	glyceryl group in **TG**
**P**	4.28	ddd	$ROC\underline{\mathbf{H}}_2-CH(OR')-CH_2OH$	glyceryl group in **1,2-DG**
**Q**	4.93	m	$HOCH_2-C\underline{\mathbf{H}}(OR)-CH_2OH$	glyceryl group in **2-MG**
**R**	5.08	m	$ROCH_2-C\underline{\mathbf{H}}(OR')-CH_2OH$	glyceryl group in **1,2-DG**
**S**	5.27	m	$ROCH_2-C\underline{\mathbf{H}}(OR')-CH_2OR''$	glyceryl group in **TG**
**T**	5.28-5.46	m	$-C\underline{\mathbf{H}}=C\underline{\mathbf{H}}-$	**AG** and **FA**

Abbreviations: t: triplet; m: multiplet; d: doublet; AG: acyl group; TG: triacylglycerols; DG: diacylglycerols; MG: monoacylglycerols; FA: fatty acid.

[§] overlapping of multiplets of methylenic protons in the different acyl groups either in *beta*-position, or further, in relation to double bonds, or in *gamma*-position, or further, in relation to the carbonyl group; [§§] overlapping of multiplets of the *alpha*-methylenic protons in relation to a single double bond of the different unsaturated acyl groups;

[§§§] this signal shows different multiplicity if the spectrum is acquired from the pure compound or taking part in the mixture.

2.4. Quantitative determinations of products arising from lipolysis

As demonstrated in previous works (Nieva-Echevarría et al., 2014; 2015), the number of moles (N) of fatty acids (**FA**), 2- and 1-monoglycerides (**2-MG**, **1-MG**), 1,2- and 1,3-diglycerides (**1,2-DG**, **1,3-DG**), and triglycerides (**TG**) can be estimated by using the area of certain spectral signals of the standard pulse spectrum:

$N_{2\text{-}MG} = Pc^{*}A_{K}/4$ [eq.S8]

$N_{1\text{-}MG} = Pc^{*}A_{L}$ [eq.S9]

$N_{1,2\text{-}DG} = Pc^{*}(A_{I+J}\text{-}2A_{L})/2$ [eq.S10]

$N_{1,3\text{-}DG} = Pc^{*}(A_{4.04\text{-}4.38}\text{-}2A_{4.26\text{-}4.38}\text{-}2A_{L})/5$ [eq.S11]

$N_{TG} = Pc^{*}(2A_{4.26\text{-}4.38}\text{-}A_{I+J}+2A_{L})/4$ [eq.S12]

$N_{FA} = (Pc^{*}A_{2.26\text{-}2.40}\text{-}6N_{TG}\text{-}4N_{1,2\text{-}DG}\text{-}4N_{1,3\text{-}DG}\text{-}2N_{1\text{-}MG}\text{-}2N_{2\text{-}MG})/2$ [eq.S13]

where Pc is the proportionality existing between the area of the ^{1}H NMR signals and the number of protons that generate them, A_{K}, A_{L} and A_{I+J} are the areas of the corresponding signals indicated in **Table S2** and **Fig.S1**, and $A_{4.04\text{-}4.38}$, $A_{4.26\text{-}4.38}$ and $A_{2.26\text{-}2.40}$ represent the area of the signals at 4.04-4.38 ppm, 4.26-4.38 ppm and 2.26-2.40 ppm, respectively.

Although formed from total hydrolysis of TG and thus present in the *in vitro* digest, glycerol (**Gol**) is absent in the corresponding lipid extract because of its hydrophilic nature. Nevertheless, the number of moles of glycerol (**N_{Gol}**) can be estimated indirectly taking into account the stoichiometry of TG hydrolysis.

$N_{Gol} = (N_{FA}\text{-}N_{1,2\text{-}DG}\text{-}N_{1,3\text{-}DG}\text{-}2N_{2\text{-}MG}\text{-}2N_{1\text{-}MG})/3$ [eq.S14]

Likewise, the total number of moles of glyceryl structures (**NT_{GS}**) and of fatty acyl chains AG+FA (**NT_{AG+FA}**) present in the samples can be estimated by using the following equations:

$NT_{GS} = N_{TG}+N_{1,2\text{-}DG}+N_{1,3\text{-}DG}+N_{2\text{-}MG}+N_{1\text{-}MG}+N_{Gol} = Pc^{*}(A_{2.26\text{-}2.40}/6)$ [eq.S15]

$NT_{AG+FA} = 3^{*}N_{TG}+2^{*}N_{1,2\text{-}DG}+2^{*}N_{1,3\text{-}DG}+N_{2\text{-}MG}+N_{1\text{-}MG} = Pc^{*}(A_{2.26\text{-}2.40}/2)$ [eq.S16]

Using all these equations, the molar percentages of the different kinds of glycerides in relation to the total number of moles of glyceryl structures present (NT_{GS}) can be determined using the following equations:

TG% = $100N_{TG}/NT_{GS}$ [eq.S17]

1,2-DG% = $100N_{1,2\text{-}DG}/NT_{GS}$ [eq.S18]

1,3-DG% = $100N_{1,3\text{-}DG}/NT_{GS}$ [eq.S19]

2-MG% = $100N_{2\text{-}MG}/NT_{GS}$ [eq.S20]

1-MG% = $100N_{1\text{-}MG}/NT_{GS}$ [eq.S21]

Gol% = $100N_{Gol}/NT_{GS}$ [eq.S22]

Finally, to estimate the extent of lipolysis in digestion from a physiological point of view, as the complete absorption of a TG only requires its conversion into MG and two FA, the parameter **lipid bioaccessibility**, which informs about the proportions of absorbable lipidic molecules released during digestion, **(L_{BA}%)** was estimated as follows:

$$L_{BA}\% = 100(N_{1\text{-}MG} + N_{2\text{-}MG} + N_{FA})/NT_{AG+FA} \qquad \text{[eq.S23]}$$

REFERENCES

Guillén, M.D.; Ruiz, A. Formation of hydroperoxy- and hydroxyalkenals during thermal oxidative degradation of sesame oil monitored by proton NMR. *Eur. J. Lipid Sci. Technol.* **2004**, 106(10), 680–687.

Guillén, M.D.; Ruiz, A. Monitoring the oxidation of unsaturated oils and formation of oxygenated aldehydes by proton NMR. *Eur. J. Lipid Sci. Technol.* **2005**, 107(1), 36-47.

Nieva-Echevarría, B.; Goicoechea, E.; Manzanos, M.J.; Guillén, M.D. A method based on ^{1}H NMR spectral data useful to evaluate the hydrolysis level in complex lipid mixtures. *Food Res. Int.* **2014**, 66, 379-387.

Nieva-Echevarría, B.; Goicoechea, E.; Manzanos, M.J.; Guillén, M.D. Usefulness of ^{1}H NMR in assessing the extent of lipid digestion. *Food Chem.* **2015**, 179, 182-190.

Nieva-Echevarría, B.; Goicoechea, E.; Manzanos, M.J.; Guillén, M.D. A study by ^{1}H NMR on the influence of some factors affecting lipid *in vitro* digestion. *Food Chem.* **2016**, 211, 17-26.

Versantvoort, C.H.M.; Van de Kamp, E.; Rompelberg, C. Development and applicability of an *in vitro* digestion model in assessing the bioaccessibility of contaminants from food. Report No. 320102002, **2004**, National Institute for Public Health and the Environment, Bilthoven, The Netherlands. Available from (Accessed November 9th 2021): http://www.rivm.nl/bibliotheek/rapporten/320102002.pdf.

Versantvoort, C. H. M.; Oomen, A. G.; Van de Kamp, E.; Rompelberg, C.J.M.; Sips, A.J.A.M. Applicability of an *in vitro* digestion model in assessing the bioaccessibility of mycotoxins from food. *Food Chem. Toxicol.* **2005**, 43(1), 31-40.
